# Caspase 4 Overexpression as a Prognostic Marker in Clear Cell Renal Cell Carcinoma: A Study Based on the Cancer Genome Atlas Data Mining

**DOI:** 10.3389/fgene.2020.600248

**Published:** 2021-01-14

**Authors:** Lingfeng Meng, Zijian Tian, Xingbo Long, Tongxiang Diao, Maolin Hu, Miao Wang, Wei Zhang, Yaoguang Zhang, Jianye Wang, Yuhui He

**Affiliations:** ^1^Department of Urology, Beijing Hospital, National Center of Gerontology, Institute of Geriatric Medicine, Chinese Academy of Medical Sciences, Beijing, China; ^2^Graduate School of Peking Union Medical College, Chinese Academy of Medical Sciences, Beijing, China; ^3^Department of Urology, Peking University First Hospital, Beijing, China

**Keywords:** caspase 4, clear cell renal cell carcinoma, overexpression, prognosis, CD4 memory T cells

## Abstract

The dysregulation of caspase 4 (*CASP4*) expression is related to the occurrence, development, and outcome of many malignant tumors; however, its role in clear cell renal cell carcinoma (ccRCC) remains unclear. Herein, we investigated the expression of *CASP4* in tumor tissues and its relationship with clinical prognosis, immune infiltration, and drug sensitivity status of ccRCC patients. Oncomine and The Cancer Genome Atlas (TCGA) databases were used to determine *CASP4* mRNA expression in ccRCC patients. The correlation between *CASP4* expression and disease prognosis was evaluated using Kaplan–Meier analysis. Related pathways were obtained from TCGA database via gene set enrichment analysis (GSEA) and gene set variation analysis (GSVA). Meanwhile, genes co-expressing with *CASP4* in ccRCC were investigated. Finally, we analyzed the proportion of tumor-infiltrating immune cells (TICs) using the CIBERSORT computational method and assessed *CASP4* methylation and its relationship with drug sensitivity. Immunohistochemical analysis of 30 paired ccRCC and adjacent normal tissues confirmed the *in silico* results. *CASP4* mRNA expression in ccRCC was significantly higher than that in the normal tissues, positively correlated with clinicopathological features (clinical stage and pathological grade), and negatively correlated with patient overall survival (OS). GSEA and GSVA showed that the genes in the *CASP4*-high expression group were primarily enriched in immune-related activities. Moreover, CIBERSORT analysis of TIC proportions revealed that activated CD4 memory T cells were positively correlated with *CASP4* expression. Notably, methylation analysis revealed that the abnormal upregulation of *CASP4* might be caused by hypomethylation. Finally, we found that the abnormal expression of *CASP4* may be related to tumor drug resistance. Overall, our study shows that *CASP4* is overexpressed in ccRCC and is an important factor affecting disease prognosis. Hence, *CASP4* may serve as a potential prognostic biomarker and therapeutic target in ccRCC.

## Introduction

Kidney cancer is one of the most common malignancies of the urinary system, accounting for over 100,000 annual deaths worldwide, of which renal cell carcinoma (RCC) is responsible for more than 90% (Bray et al., 2018). In fact, RCC ranks seventh and ninth in the incidence of malignant tumors among men and women, respectively (Cho et al., 2011). Epidemiological investigations have shown that RCC is the second most common tumor of the genitourinary system in China after bladder tumors (Pang et al., 2016). Clear cell RCC (ccRCC) is the most common pathological form of RCC, accounting for 75–80% of cases, with a higher incidence in males than in females (Cohen and Mcgovern, 2005). Surgery is the first-line treatment for ccRCC in the early stage, with a 5-year survival rate after early surgery being approximately 70%. However, approximately 30% of patients show relapse within five years after surgery (di Martino et al., 2018). Unfortunately, early diagnosis is difficult, resulting in many patients requiring radical nephrectomy upon diagnosis. Moreover, ccRCC is insensitive to conventional radiotherapy and chemotherapy and has a poor prognosis following metastasis. Hence, it is imperative to elucidate the molecular mechanisms underlying ccRCC occurrence and development. Moreover, the identification of novel molecular diagnostic markers can facilitate early diagnosis and expand our understanding of disease progression while also providing potential targets for the treatment of ccRCC.

Using data from multiple ccRCC patient datasets, Xie et al. (2020) found that patients showing high expression of *P4HB* had a poor prognosis. Meanwhile, based on the analysis of multiple datasets together with *in vitro* cell experiments, Li et al. (2020) speculated that *PATJ* may be a potential therapeutic target of ccRCC. These results demonstrate the validity of utilizing gene database analysis (based on the new second-generation sequencing methods) to identify clinical cancer biomarkers.

In the human genome, the caspase (aspartate-specific cysteinyl proteinase) family contains at least 12 members (including *CASP1*, *CASP3*, and *CASP4*), which are involved in several important physiological processes, such as apoptosis, growth, necrosis, and inflammation (Van Opdenbosch and Lamkanfi, 2019). Among them, caspase 4 (*CASP4*) has been associated with many diseases, including inflammatory bowel disease (Flood et al., 2015), Alzheimer’s disease (Kajiwara et al., 2016), Parkinson’s disease (Arai et al., 2006), and various malignancies (Scapoli et al., 2011; Nilsson, 2013; Schulten et al., 2016; Papoff et al., 2018). However, the relationship between the expression of *CASP4* and the clinical prognosis of ccRCC patients remains unclear. Notably, the function of *CASP4* in autophagy and its effect on the growth and migration of tumor cells indicate that it is a potential target for cancer therapy. In fact, caspases, in general, are believed to serve as key factors for the development of effective anticancer therapies. Herein, the expression of *CASP4* in tumor tissues and its relationship with the clinical prognosis of ccRCC patients was investigated.

## Materials and Methods

### Study Aim, Design, and Setting

We evaluated the expression of *CASP4* in tumor tissues to determine its role in patients with ccRCC. More specifically, we explored the expression and functional enrichment of tumor-infiltrating immune cells (TICs) and the prognostic role of *CASP4* in 611 patients with ccRCC from The Cancer Genome Atlas (TCGA) database. Simultaneously, the relationship between the *CASP4* expression levels and methylation and drug sensitivity was explored. We also recruited 30 patients who had previously undergone radical nephrectomy and were diagnosed with ccRCC at the Beijing Hospital to verify our results. All protocols were approved by the Research Ethics Committee of Beijing Hospital, and written informed consent was obtained from all participants (2020BJYYEC-192-01).

### Data Sources

Both the mRNA expression profiles and corresponding clinical information of patients with ccRCC were obtained from TCGA. As of January 16, 2020, the platform contained 539 ccRCC and 72 adjacent non-tumor tissues. Additionally, Xie et al. (2019) used the Gene Expression Omnibus database (GSE29609 datasets, GSE22541 datasets, and GSE3 datasets) and TCGA database to obtain a total of 629 kidney renal clear cell carcinoma cases with gene expression profile data and clinical follow-up information; this information was used to develop an online analysis tool (OSkirc, http://bioinfo.henu.edu.cn/KIRC/KIRCList.jsp), which was used in the current study to analyze *CASP4* expression further.

Moreover, 30 patients with ccRCC who underwent radical nephrectomy at the Beijing Hospital from December 2012 to April 2019 were selected for the study. Immunohistochemical staining was performed on their tissue samples, and survival information was followed up through telephone conversations.

### Differential Expression of *CASP4* and Establishment of Survival and Risk Curves

We used the Limma R package to analyze the differential expression of *CASP4* in ccRCC and normal tissue samples. The results were visualized as a box diagram. We also divided patients with ccRCC from TCGA database into low- and high-expression groups based on median *CASP4* expression and generated Kaplan–Meier curves to compare survival between groups. Risk curves were generated for both groups. Of note, Beijing Hospital patients with ccRCC were also divided into low- and high-risk groups according to their median immunohistochemical score (H-score), and Kaplan–Meier curves were generated to compare the differences.

### Correlations Between *CASP4* Expression, Clinicopathological Parameters, and Molecular Characteristics of ccRCC Patients

To analyze the relationship between the *CASP4* expression and the clinicopathological characteristics of ccRCC patients, we used patient age, sex, pathological grade, and clinical stage as classification variables.

In addition, we further explored whether *CASP4* expression can be independent of other clinical variables, using univariate and multivariate Cox regression analysis, clinical information as the independent variable, and survival time as the dependent variable; The 95% confidence interval and hazard ratio were calculated.

Gene set variation analysis (GSVA) is a pathway-based analysis method that provides each sample with an overall pathway or gene set activity score. We used the median *CASP4* expression to divide patients into high- and low-risk groups and then used the GSVA R package to identify and visualize their most relevant pathways. In addition, we used the gene set enrichment analysis (GSEA) software v4.0 to analyze patients in high- and low-risk groups to explore which functions or pathways of *CASP4* play a role in tumorigenesis. To explore the possible molecular mechanisms associated with *CASP4*, we identified genes co-expressed with *CASP4* in ccRCC patients. To obtain high-dimensional information, we used the “ggplot2” and “circlize” R packages to visualize co-expressed genes and their relationships.

### Correlation Between *CASP4* Expression and TICs

To identify the relationship between *CASP4* expression and the immune microenvironment, we used the CIBERSORT algorithm to analyze the proportion of tumor-infiltrating immune subsets. The CIBERSORT R package was downloaded from https://cibersortx.stanford.edu/, with a document using a set of 22 immune cell reference profiles to derive a signature matrix which can be applied to the ccRCC samples in order to determine relative proportions of immune cells. The *P* value was calculated for each sample. If *P* > 0.05, the proportion of tumor-infiltrating immune subsets calculated using this algorithm is inaccurate; hence, all samples with *P* > 0.05 were excluded, and only 539 tumor samples, with *P* < 0.05, were analyzed.

We divided the ccRCC patients into high- and low-expression groups based on the median *CASP4* expression levels and performed differential analysis to identify differentially abundant immune cells. We further used correlation analysis to identify immune cells related to the expression of *CASP4*. The results obtained with the two application methods were overlaid.

### Correlation Between *CASP4* Expression and Methylation and Drug Sensitivity Patterns

The human disease methylation database (DiseaseMeth 2.0, http://bio-bigdata.hrbmu.edu.cn/diseasemeth/) integrates methylation group data from microarrays and sequencing techniques and annotates the DNA methylation status in human diseases. We used this website to compare and visualize the methylation levels of *CASP4* in ccRCC and para-cancerous tissues.

In addition, we downloaded data of different cancer cell lines from the NCI-60 database^1^. The Pearson correlation was used to explore the relationship between *CASP4* expression and drug sensitivity. Here, the analyses focused only on 263 drugs (approved by the Food and Drug Administration or undergoing clinical trials).

### Statistical Analysis

Statistical analyses were performed using the SPSS software v. 22.0 (Chicago, IL, United States) and the R package v. 3.6.1^2^. Wilcox rank-sum test was performed to analyze the differential gene expression between tumor and normal tissues. ccRCC patients of different datasets were categorized into high-risk and low-risk groups based on the expression level of *CASP4* or the median of histochemistry score, while the overall survival (OS) of the patients was analyzed using the Kaplan–Meier method. Univariate and multivariate Cox regression analyses were performed to elucidate the relationship between gene expression and OS. In addition, the “ggplot2” package in R software was used to explore the genes co-expressed with *CASP4*. The correlation between gene expression and drug sensitivity was obtained by calculating the Pearson correlation coefficient. Statistical significance was defined as *P* < 0.05.

## Results

### *CASP4* Is Highly Expressed in ccRCC

**FIGURE 1:**
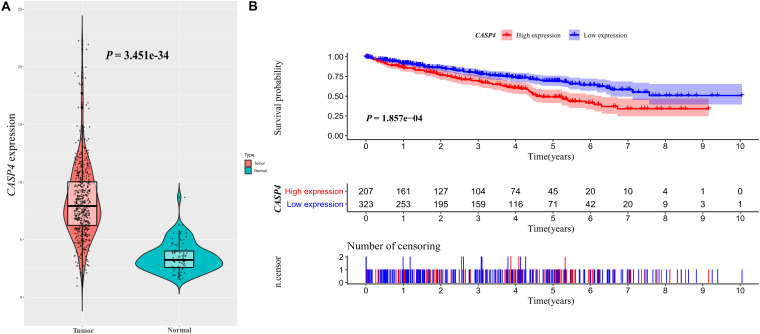
*CASP4* expression in clear cell renal cell carcinoma (ccRCC) patients and its relationship with prognosis: Data retrieved from The Cancer Genome Atlas database. **(A)** Difference in the expression of *CASP4* between ccRCC and normal tissues. **(B)** The overall survival (OS) of patients with high *CASP4* expression was significantly lower than that of patients with low *CASP4* expression. The shaded area represents the 95% confidence interval.

**FIGURE 2:**
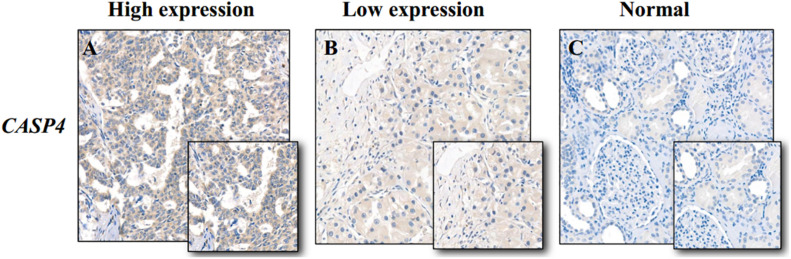
Immunohistochemistry staining of caspase 4 (*CASP4*) in clear cell renal cell carcinoma (ccRCC) and adjacent normal tissues. **(A)** High *CASP4* expression in ccRCC tissues; **(B)** low *CASP4* expression in ccRCC tissues; and **(C)** low *CASP4* expression in adjacent normal tissues.

We used the Oncomine database to determine the *CASP4* mRNA expression levels in ccRCC versus normal tissues. The *CASP4* mRNA levels were significantly higher in ccRCC than in normal tissues (Supplementary Figure 1, *P* < 0.001). These data were consistent with those obtained in the context of TCGA database (Figure 1A). Moreover, the protein expression data (based on immunohistochemistry staining) for the 30 pairs of ccRCC and adjacent normal tissue samples collected at our center were consistent with the mRNA expression data (Figure 2).

### High *CASP4* Expression Is Associated With Worse OS in ccRCC Patients

**FIGURE 3:**
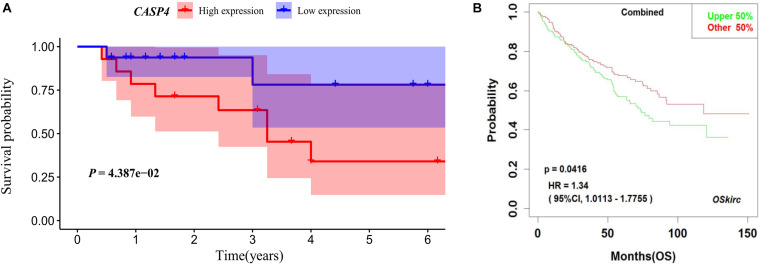
Survival of patients with different caspase 4 (*CASP4*) expression levels based on an external dataset. **(A)** Based on the H-Scores for *CASP4*, the overall survival of high-risk patients was significantly lower than that of low-risk patients. The shaded area represents the 95% confidence interval. **(B)** Kaplan–Meier analysis of patients with high and low expression of *CASP4* in the OSkirc.

Our analysis of TCGA database revealed that the survival of ccRCC patients with high *CASP4* mRNA expression was shorter than that of patients with low expression (*P* < 0.001, Figure 1B). We also visualized the distribution of high and low *CASP4* expression in patients with ccRCC and the number of patients in the high and low expression groups (Supplementary Figure 2). Samples from our center were further divided into high- and low-expression groups based on the median H-score; Kaplan–Meier curves were subsequently generated to compare the OS. The H-score was calculated as described previously (Yeo et al., 2015). Importantly, Kaplan–Meier analysis showed that *CASP4* upregulation was closely related to the OS of ccRCC patients (*P* < 0.05, Figure 3A). Similar results were observed for the OSkirc-related data (*P* < 0.05, Figure 3B).

### Correlation Between *CASP4* Expression and ccRCC Patient Clinicopathological Parameters

**FIGURE 4:**
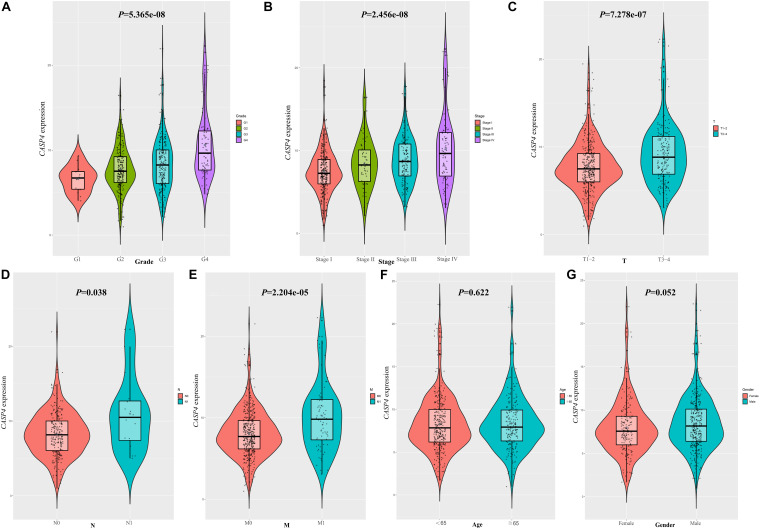
Clinicopathological significance of caspase 4 (*CASP4*) expression. Expression of *CASP4* as a function of **(A)** pathological grade, **(B)** clinical stage, **(C)** T stage, **(D)** N stage, **(E)** M stage, **(F)** age, and **(G)** sex.

We found that the expression of *CASP4* increased with an increasing pathological grade (*P* < 0.001, Figure 4A). Similar results were observed for the clinical stages (*P* < 0.001, Figure 4B). Moreover, the expression of *CASP4* was higher in T3–4 versus T1–2 disease (*P* < 0.001, Figure 4C), N1 versus N0 (*P* = 0.038, Figure 4D), and M1 versus M0 (*P* < 0.001, Figure 4E). Meanwhile, no difference in *CASP4* expression was observed between older and younger patients (*P* = 0.622, Figure 4F) or between male and female patients (*P* = 0.052; Figure 4G).

Univariate and multivariate Cox regression analysis showed that *CASP4* expression was one of the factors affecting patient survival; however, this expression was not an independent predictor of survival (Supplementary Figure 3). This suggests that *CASP4* may influence the survival of patients by affecting other factors.

### *CASP4* Functional Enrichment and Co-expression Analyses

**FIGURE 5:**
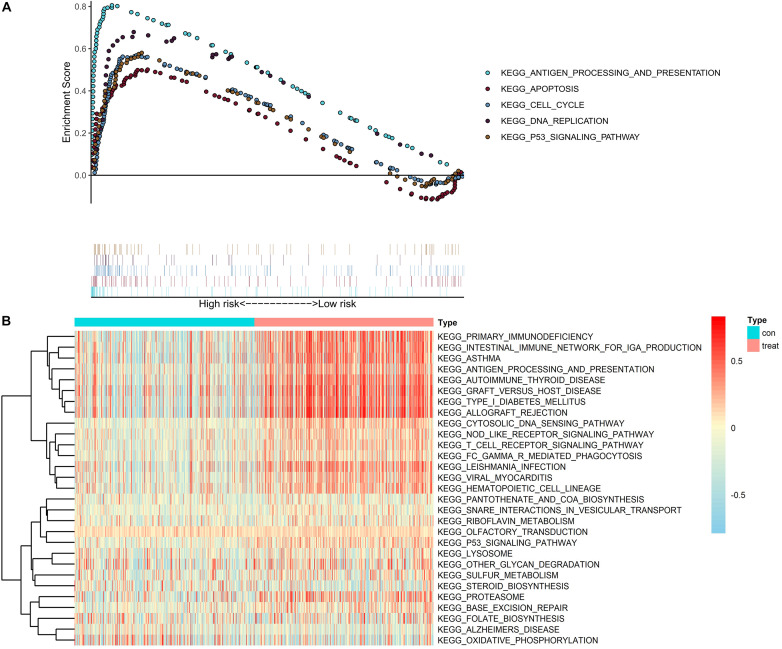
Gene set enrichment analysis and gene set variation analysis of *CASP4* expression in data retrieved from The Cancer Genome Atlas dataset. **(A)** Gene set enrichment analysis and **(B)** gene set variation analysis. Only signaling pathways with a log_2_ (fold-change) > 0.2 are shown.

To further explore the potential function of *CASP4* in ccRCC, we analyzed RNA-seq data from patients with ccRCC (retrieved from TCGA database) using GSEA and GSVA. According to the median *CASP4* expression levels in TCGA dataset, ccRCC samples were divided into high- and low-expression groups. Notably, both analyses showed that gene sets with higher scores were significantly enriched in immune- and cell cycle-related pathways (Figures 5A,B).

**FIGURE 6:**
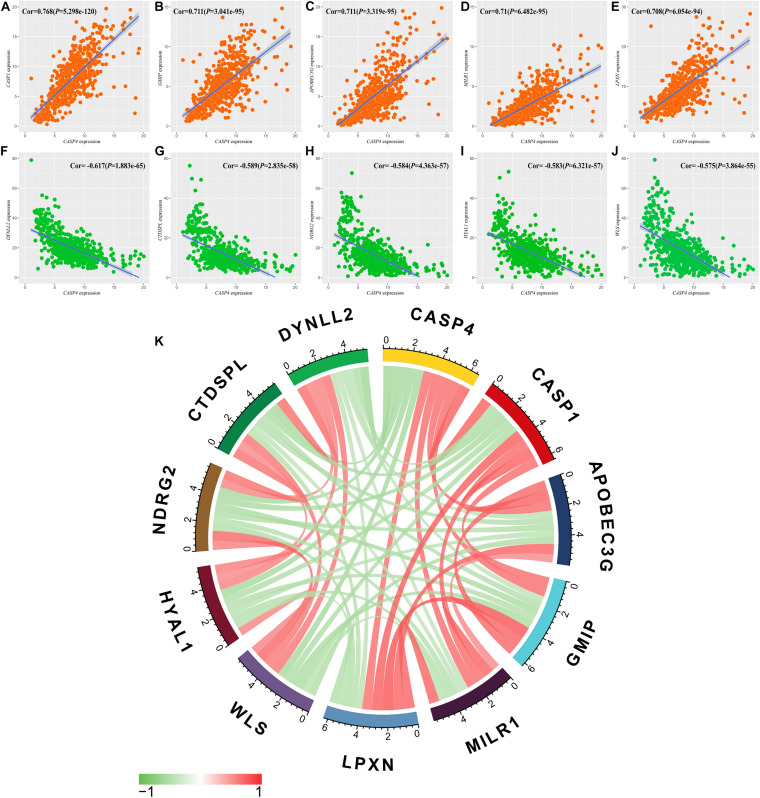
Relationships between *CASP4* expression and that of multiple genes. **(A)** *CASP1*; **(B)** *GMIP*; **(C)** *APOBEC3G*; **(D)** *MILR1*; **(E)** *LPXN*; **(F)** *DYNLL2*; **(G)** *CTDSPL*; **(H)** *NDRG2*; **(I)** *HYAL1*; **(J)** *WLS*; and **(K)** Circle diagram of the expression relationship between genes.

We also identified genes co-expressed with *CASP4* in ccRCC using a false discovery rate of <0.001 and a log_2_ (fold-change) > 0.5 as the standard. Genes with the strongest positive (*n* = 5) and negative correlations (*n* = 5) were selected. The five genes showing the strongest positive correlations were *CASP1* (Cor = 0.768, Figure 6A), *GMIP* (Cor = 0.711, Figure 6B), *APOBEC3G* (Cor = 0.711, Figure 6C), *MILR1* (Cor = 0.71, Figure 6D), and *LPXN* (Cor = 0.708, Figure 6E). The five genes showing the strongest negative correlations were *DYNLL2* (Cor = −0.617, Figure 6F), *CTDSPL* (Cor = −0.589, Figure 6G), *NDRG2* (Cor = −0.584, Figure 6H), *HYAL1* (Cor = −0.583, Figure 6I), and *WLS* (Cor = −0.575, Figure 6J). Finally, we constructed a co-expression circle map to visualize the relationships among these genes (Figure 6K).

### *CASP4* Expression Affects Immune Activity of the Tumor Microenvironment

**FIGURE 7:**
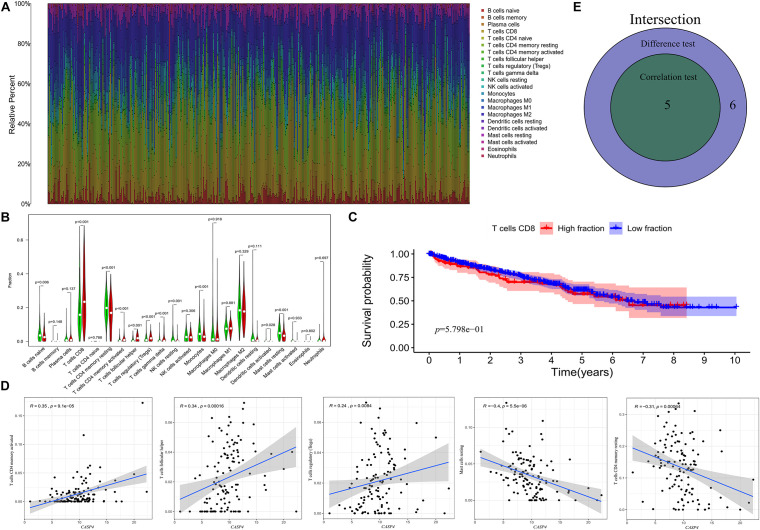
Distribution of tumor-infiltrating immune cells (TICs) in tumor samples and their correlation with *CASP4* expression. **(A)** Proportion of 22 types of TICs in ccRCC tumor samples. **(B)** Ratio differentiation of the 22 types of immune cells in patients with high and low expression of *CASP4*. **(C)** The median value of the fraction of CD8 T cells showed no significant difference in the survival curves of the high and low fraction groups. The shaded area represents the 95% confidence interval. **(D)** Correlation of the proportion of five types of TICs and the expression of *CASP4*; **(E)** Venn plot is shown.

We then assessed the proportion of tumor-infiltrating immune subsets using the CIBERSORT algorithm and constructed 22 immune cell profiles for ccRCC samples (Figure 7A). The fraction of CD8 T cells was significantly higher than that of other immune cells (Figure 7B); therefore, we further divided CD8 T cells into high and low fraction groups, although the survival curve showed no significant differences (Figure 7C).

Correlation analyses showed that five types of TICs were correlated with the expression of *CASP4* (Figures 7D,E). Among them, activated CD4 memory T cells, follicular helper T cells, and regulatory T cells (Tregs) were positively correlated with *CASP4* expression, whereas resting CD4 memory T cells and resting mast cells were negatively correlated with *CASP4* expression. These results suggest that *CASP4* expression affects immune activity in the tumor microenvironment (TME).

### *CASP4* Expression Is Associated With Methylation and Cancer Cell Sensitivity to Chemotherapy

**FIGURE 8:**
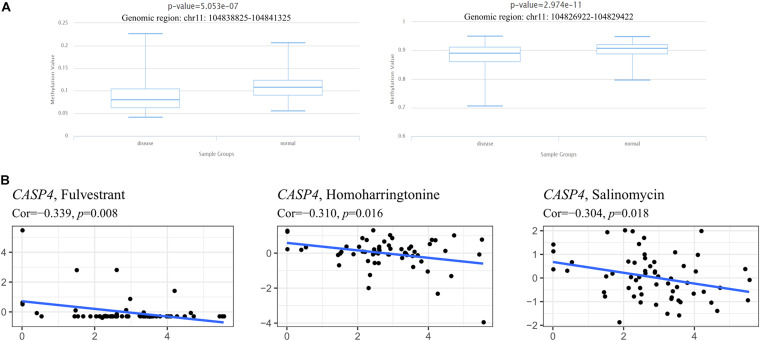
Methylation analysis of the *CASP4* gene and its relationship with drug sensitivity. Detection of methylation levels in clear cell renal cell carcinoma and para-cancerous normal tissues based on DiseaseMeth 2.0 **(A)**. Using NCI-60 cell line-derived data, Pearson correlation tests were performed to analyze the relationship between the expression of *CASP4* and drug sensitivity **(B)**.

We also evaluated the relationship between *CASP4* expression and its methylation status to determine the potential mechanism underlying its aberrant upregulation in ccRCC tissues. When comparing the methylation level of *CASP4* in ccRCC tissues and para-cancerous tissues using the DiseaseMeth 2.0 database, we found that *CASP4* corresponds to two different genomic regions in the database. In both genomic regions, the average methylation level of *CASP4* in ccRCC was significantly lower than that in para-cancerous tissues (Figure 8A).

Meanwhile, we also investigated the relationship between *CASP4* expression and drug sensitivity and sorted the three most relevant results based on their *P*-value (lowest to the highest; Figure 8B). We observed that as *CASP4* expression increased, the sensitivity of cells to chemotherapeutic drugs decreased.

## Discussion

Approximately, 2–3% of all human cancers are ccRCC, with the morbidity and mortality of ccRCC increasing significantly over the past 20 years (Ferlay et al., 2013). Moreover, an estimated 300,000 people are diagnosed with ccRCC each year, which was expected to increase by 22% in 2020 (Ferlay et al., 2015). As a complex and heterogeneous disease, the pathogenesis of ccRCC is not completely understood. Although many studies have been carried out using microarrays and RNA-seq to identify new biomarkers and therapeutic targets, many inconsistencies have been reported regarding differentially expressed genes in ccRCC. Therefore, it is necessary to find a reliable and relatively simple method to assess disease prognosis accurately.

The prognostic significance of *CASP4* overexpression in tumors remains controversial. For instance, Wang et al. (2019) reported that a higher expression of *CASP4* was associated with a better OS in patients with gastric cancer. Similarly, Shibamoto et al. (2017) reported that *CASP4* may be a tumor suppressor gene in esophageal squamous cell carcinoma. However, Terlizzi et al. (2018) found that *CASP4* overexpression was associated with poor prognosis in patients with non-small cell lung cancer; in fact, *CASP4* was defined as a new biomarker for the diagnosis and prognosis of non-small cell lung cancer. However, the conclusions of these studies require further verification. Hence, the results of the above studies indicate a significant heterogeneity in *CASP4* expression among different tumor types with altered *CASP4* expression generally related to patient OS, and the direction of the association depends on the type of cancer.

It has been suggested that CASPs may be the key to develop more effective anticancer therapies (Frejlich et al., 2013). The important role of these proteins in various diseases has been identified and their substrates are considered targets of anticancer drugs; the goal is to induce the death of malignant tumor cells, acting upstream of CASPs (Frejlich et al., 2013; Julien and Wells, 2017). However, the prognostic significance of *CASP4* in ccRCC has not yet been reported. We found that the expression levels of *CASP4* in ccRCC samples were significantly higher than that in normal samples and that the OS of patients with high *CASP4* mRNA levels was worse than that of patients with low expression of *CASP4* mRNA. These conclusions were reached based on external data sets and experimental data. Importantly, these findings show that the levels of this protein in ccRCC tumor tissues can act as not only a diagnostic tool but also a prognostic biomarker, a much-needed tool for the diagnosis and monitoring of ccRCC patients. Therefore, we believe that by developing drugs targeting *CASP4* and the reducing its expression in ccRCC patients, the prognosis of ccRCC patients may be improved.

Previous studies have reported the relationship between the expression of *CASP4* and the clinicopathological parameters of tumor patients. Of note, the expression of *CASP4* was not related to age or sex (Shibamoto et al., 2017; Terlizzi et al., 2018). This was also confirmed in the current study (*P* = 0.622, *P* = 0.052). However, our data did show that *CASP4* expression increases with advanced TNM stage and pathological grade. Moreover, high expression of *CASP4* often indicates a very poor prognosis for patients.

To further elucidate the role of *CASP4* in ccRCC, we used GSEA and GSVA to identify important pathways via analysis of publicly available data. We found that *CASP4* upregulation was related to the immune response and signaling pathways involved in apoptosis. Importantly, inflammatory caspases, including *CASP4*, play a key role in the immune response as they can promote the fusion of phagosomes and lysosomes of pathogenic bacteria, prevent pathogens from replicating in cells, and promote the maturation and secretion of inflammatory cytokines (Man and Kanneganti, 2016; Papoff et al., 2018; Songane et al., 2018).

Genes co-expressed with *CASP4* were also identified in this study, of which we analyzed the two with the strongest positive and negative correlations, namely *CASP1* and *DYNLL2*. CASP1 and CASP4 belong to the same caspase subfamily. Interestingly, Dong et al. (2009) reported in a multicenter case-control study that three single-nucleotide polymorphisms (SNPs) in CASP1, 4, 5, and 12 are associated with an increased risk of renal cancer, while one is associated with a reduced risk of renal cancer; however, the four SNPs exhibited minimal correlations with each other. Nevertheless, Dong et al. (2009) did not explore the relationship associated with the co-expression of these genes. Combining the earlier findings and conclusions of our study, we speculate that the members of this caspase subfamily may exert a synergistic effect on the occurrence and development of renal cancer. Moreover, previous studies have reported that DYNLL2 is associated with drug resistance to sorafenib in patients with hepatocellular carcinoma (Liu et al., 2019). Sorafenib was approved in China in 2006 and has been used as a first-line treatment for metastatic RCC with reliable results (Zhang et al., 2015). The negative correlation between *CASP4* and *DYNLL2* suggests that it may indirectly interfere with the expression of *DYNLL2* to improve sorafenib sensitivity in patients; however, this hypothesis required further verification.

In recent years, immunotherapy has improved the treatment of advanced RCC. Indeed, by the end of 2015, immune checkpoint inhibitors were approved as second-line treatments for advanced RCC, effectively propeling the treatment of advanced RCC to the immunotherapy era (Sadreddini et al., 2019). Considering the potential role of the association between the immune response and *CASP4* in ccRCC found in this study, as well as the reliable results obtained for immunotherapy in RCC, we further explored the infiltration of immune cells in ccRCC tumor tissues and the immune cells related to *CASP4*. CIBERSORT analysis of the proportion of TICs showed a positive correlation between *CASP4* and activated CD4 memory T cells in patients with ccRCC. However, contradictory theories exist regarding the role of CD4+ T cells in cancer. For example, it has been proposed that infiltrating CD4+ T cells promote the proliferation of RCC cells via regulating the TGF β1/YBX1/HIF2α signaling pathway (Wang et al., 2018). Meanwhile, activated CD4+ memory T lymphocytes and CD4+ helper T cells can target antigenic tumor cells, inhibit tumor growth, and have a positive regulatory role in anti-tumor immunity (Vesely et al., 2011; Hirschhorn-Cymerman et al., 2012). It is currently believed that the production of a T cell-mediated immune response is the basis of effective anti-tumor immunotherapy. Specifically, the cytotoxic properties of CD8+ T cells make them the main cells associated with the control of immunogenic tumor cell growth, whereas CD4+ T cells function primarily by secreting various cytokines (Zou and Chen, 2008). Therefore, through analysis of the TIC proportions in ccRCC patients, it is suggested that *CASP4* may be involved in the maintenance and regulation of immune activity in the TME, which could provide new therapeutic targets and insights for the development of effective ccRCC immunotherapies.

Recent studies have also shown DNA methylation is associated with many malignant tumor types, including urinary system tumors (Negraes et al., 2008). Interestingly, compared to the para-cancerous samples, *CASP4* was hypomethylated in ccRCC tissues, which was consistent with the observed upregulation of *CASP4* expression in ccRCC. Based on the role of methylation on gene expression and the significant increase in *CASP4* expression in ccRCC tissues, we speculate that *CASP4* might have been upregulated due to demethylation in its promoter region. Similar conclusions were drawn by Zhu and Li (2018), who concluded that abnormal methylation levels of the promoter region of *CASP4* are related to the occurrence and development of breast cancer. Furthermore, we used the NCI-60 database to explore the relationship between *CASP4* expression and drug resistance in tumor cells. The results showed that with increased *CASP4* expression, a corresponding increase was observed in resistance to certain chemotherapeutic drugs, including fulvestrant, homoharringtonine, and salinomycin. Among them, fulvestrant is the only selective estrogen receptor degrader approved for clinical use as a monotherapy for first- and second-line treatment in the context of estrogen receptor-positive advanced breast cancer (Blackburn et al., 2018; Soleja et al., 2019). Meanwhile, homoharringtonine is primarily administered to treat acute myelogenous leukemia (Chen et al., 2019); and salinomycin can be used in a variety of cancers, including RCC (Kim et al., 2017; Jiang et al., 2018; Liu et al., 2018). These data indicate that *CASP4* may play a role in the sensitivity or resistance of tumor cells to drug therapy and can be used as a therapeutic target to overcome drug resistance or increase drug sensitivity.

Previous studies have shown that CASP4 protein is related to immunity and inflammation and involved in the coordination of cellular processes, including cell homeostasis, inflammation, and apoptosis (Krause et al., 2018). Our study showed that *CASP4* is also closely related to the occurrence and development of tumors. Specifically, abnormal *CASP4* expression may be involved in the occurrence, development, and metastasis of tumors, in addition to affecting drug sensitivity. This study suggests that the analysis of *CASP4* in tumor cells should overcome the constraints of traditional concepts, not only limited to the role of inflammatory bodies in the immune system but also focusing on the interdependence or mutual restriction between *CASP4*, upstream regulatory factors, and downstream effector molecules, as well as on cell death.

Certain limitations were noted in this study. First, it is a retrospective study; hence, selection bias is inevitable. Further, the sample size was relatively small, and the population diversity was poor. Additionally, we used only immunohistochemistry (a semi-quantitative method) to detect the expression of CASP4 protein. Finally, we have not clarified the potential mechanism through which *CASP4* participates in ccRCC. Therefore, future *in vitro* and *in vivo* studies are warranted to explore the detailed mechanism underlying the association between *CASP4* expression and ccRCC occurrence and development, to provide direction for further exploration into the mechanism associated with the effects of this gene in cancer.

## Conclusion

Overall, our study showed that *CASP4* expression is upregulated in ccRCC patients and is significantly associated with a late clinical stage, a high pathological grade ccRCC, as well as a low survival rate. Importantly, this study highlights the relevance of *CASP4* in the occurrence and development of ccRCC, particularly with respect to the TME and drug resistance. Thus, *CASP4* may serve as a useful prognostic marker for patients with ccRCC, relevant not only for diagnosis but also for monitoring treatment efficacy. Of note, our data suggest that *CASP4* is a potential target for the treatment of ccRCC, which must be verified by future studies.

## Data Availability Statement

The original contributions presented in the study are included in the article/Supplementary Material, further inquiries can be directed to the corresponding authors.

## Ethics Statement

The studies involving human participants were reviewed and approved by Research Ethics Committee of Beijing Hospital. The patients/participants provided their written informed consent to participate in this study.

## Author Contributions

LM and ZT drafted and revised the manuscript. XL, TD, MH, MW, WZ, YZ, JW, and YH contributed equally to the study concerning patient recruitment, data collection, and data analysis. All authors reviewed and approved the final version of the manuscript.

## Conflict of Interest

The authors declare that the research was conducted in the absence of any commercial or financial relationships that could be construed as a potential conflict of interest.

## Footnotes

1 https://discover.nci.nih.gov/cellminer/home.do

2 https://www.r-project.org/

## Supplementary Material

Supplementary Figure 1 Prediction of high expression levels of *CASP4* mRNA in ccRCC using the Oncomine database.

Supplementary Figure 2 Visualization of *CASP4* expression in clear cell renal cell carcinoma (ccRCC) patients. **(A)** Distribution of the *CASP4* expression values. **(B)** Survival status of patients with high and low expression levels of *CASP4*.

Supplementary Figure 3 Univariate **(A)** and multivariate **(B)** COX regression analysis of the prognostic effect of *CASP4* expression in clear cell renal cell carcinoma (ccRCC) patients.

## Abbreviations

*CASP4*: Caspase 4
ccRCC: clear cell renal cell carcinoma
GSEA: gene set enrichment analysis
GSVA: gene set variation analysis
OS: overall survival
RCC: renal cell carcinoma
TCGA: The Cancer Genome Atlas
TIC: tumor-infiltration of immune cell
TME: tumor microenvironment.
