# Supplementary figures and images for: Caspase 4 Overexpression as a Prognostic Marker in Clear Cell Renal Cell Carcinoma: A Study Based on the Cancer Genome Atlas Data Mining
Source: Front Genet. 2021 Jan 14;11:600248. doi: 10.3389/fgene.2020.600248 (PMC7874118; doi:10.3389/fgene.2020.600248)

# T cells CD8( $p=0.533$ )

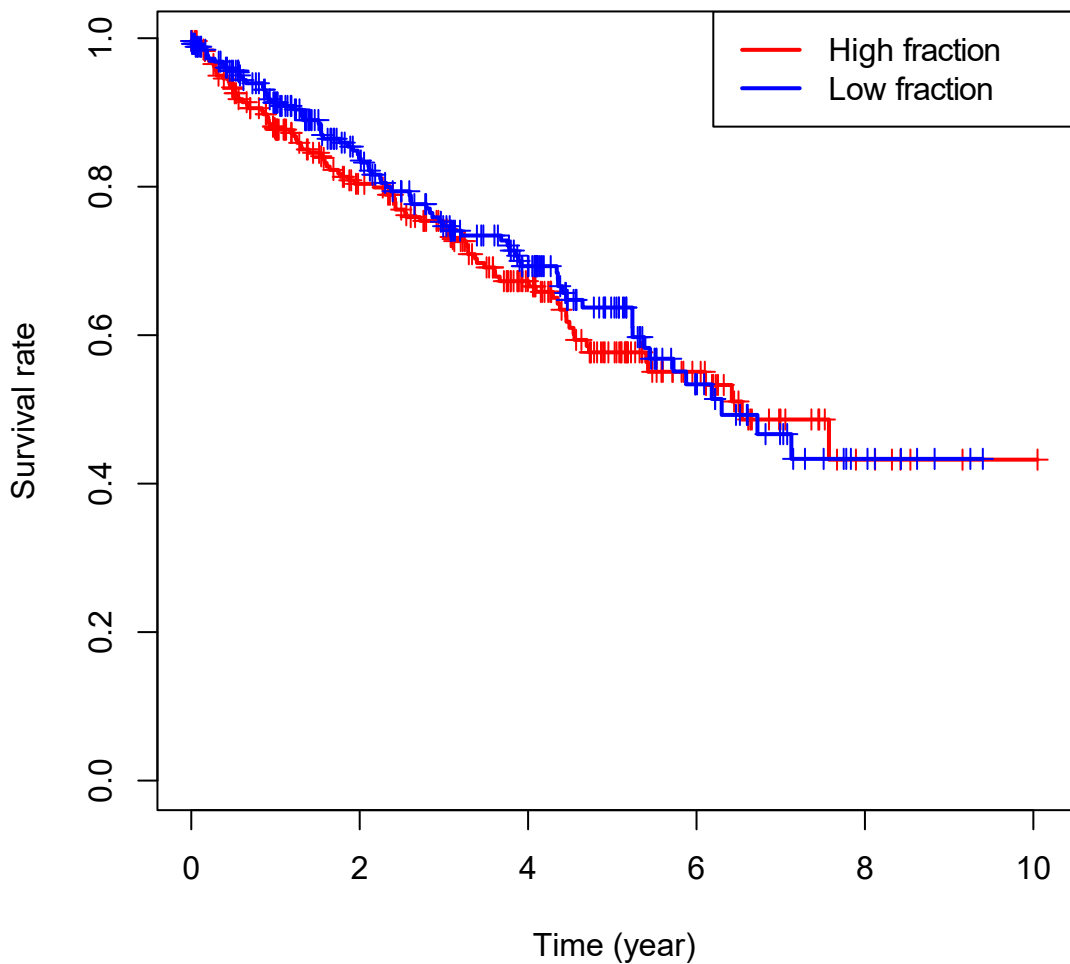

Supplement: Supplementary file 1 [file Data_Sheet_2.ZIP › CD8/survival.pdf]

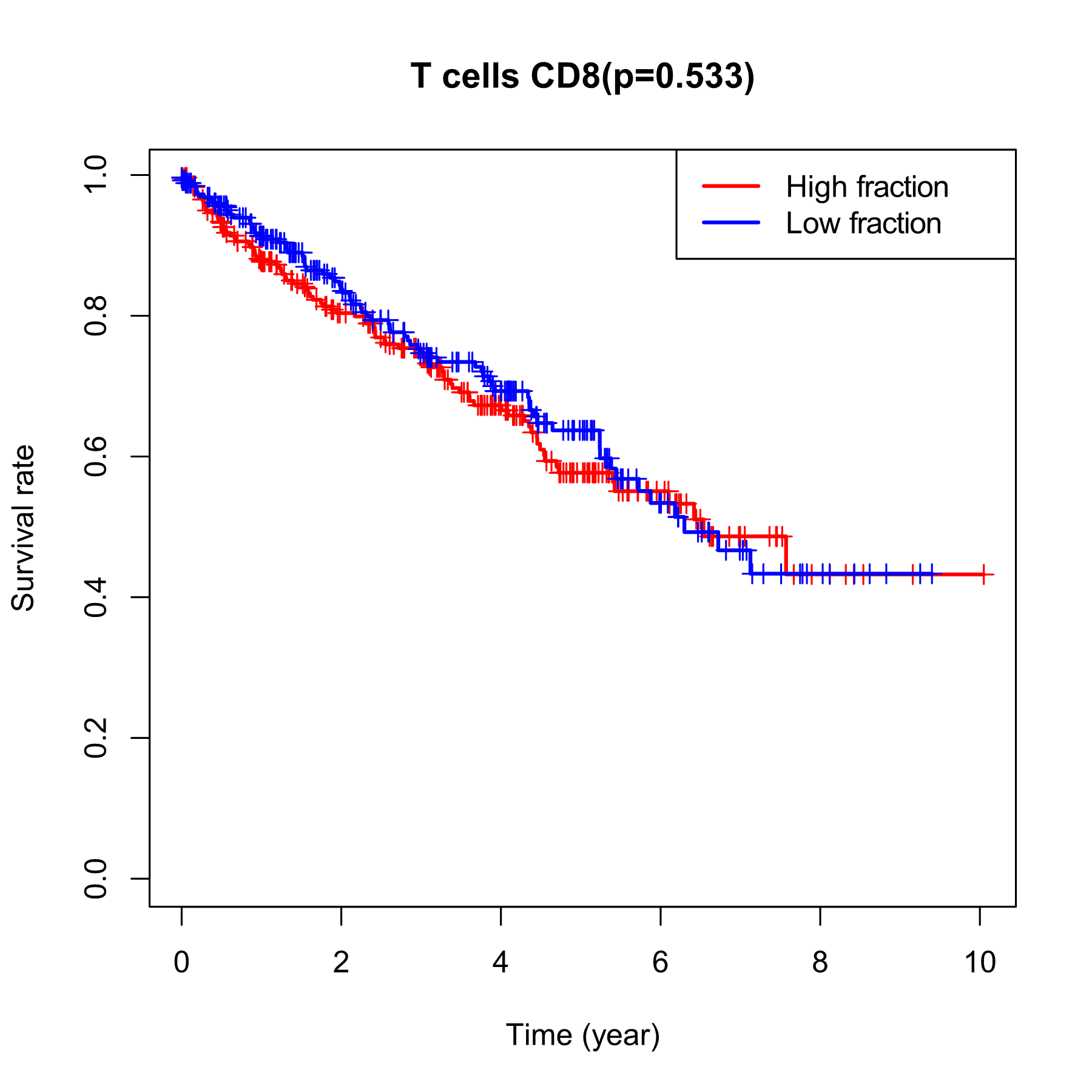

Supplement: Supplementary file 1 [file Data_Sheet_2.ZIP › CD8/survival_1.tif]
