# Supplementary material for: Caspase 4 Overexpression as a Prognostic Marker in Clear Cell Renal Cell Carcinoma: A Study Based on the Cancer Genome Atlas Data Mining
Source: Front Genet. 2021 Jan 14;11:600248. doi: 10.3389/fgene.2020.600248 (PMC7874118; doi:10.3389/fgene.2020.600248)
Supplement: Supplementary Figure 1 — Prediction of high expression levels of CASP4 mRNA in ccRCC using the Oncomine database. [file Data_Sheet_1.docx]

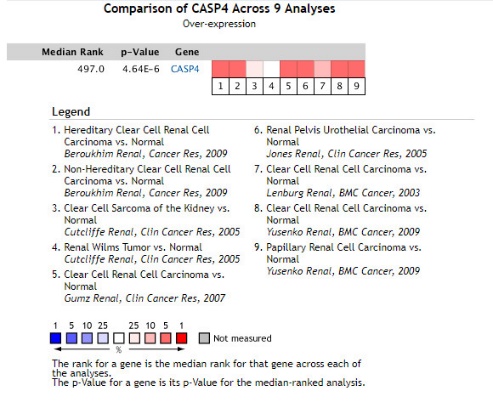
Supplementary Material

**1 Supplementary Figures**

**Supplementary Figure 1.** Prediction of high *CASP4* mRNA expression in clear cell renal cell carcinoma (ccRCC) using the Oncomine database.


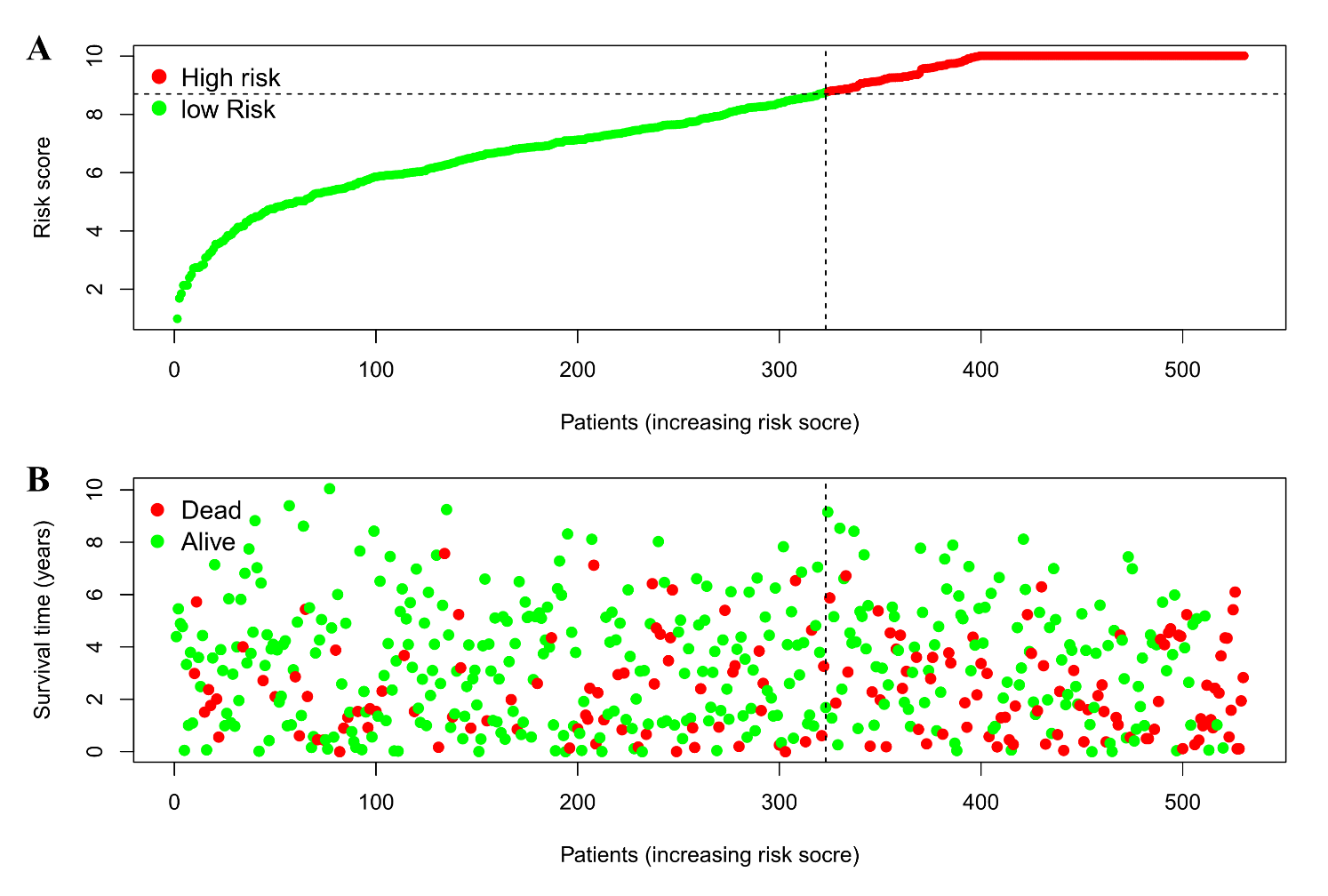


**Supplementary Figure 2.** Visualization of *CASP4* expression in clear cell renal cell carcinoma (ccRCC) patients. **(A)** Distribution of *CASP4* expression values. **(B)** Survival status of patients with high and low expression of *CASP4*.


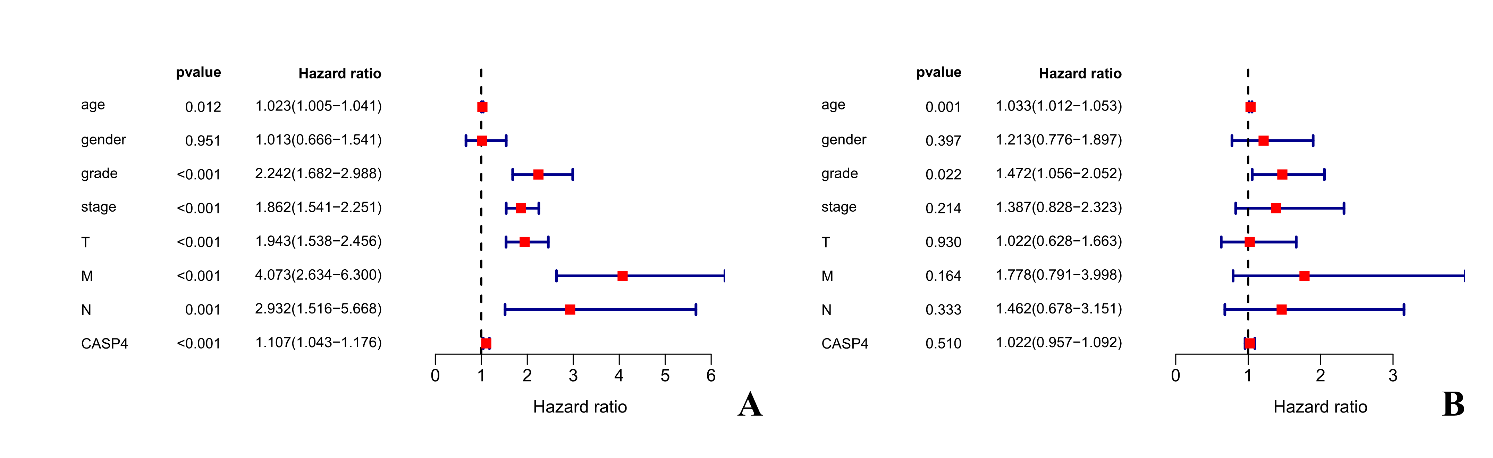


**Supplementary Figure 3.** Univariate **(A)** and multivariate **(B)** COX regression analysis of the prognostic effect of *CASP4* expression in clear cell renal cell carcinoma (ccRCC) patients.
